# Supplementary figures and images for: Mechanisms of peptide agonist dissociation and deactivation of adhesion G-protein-coupled receptors
Source: bioRxiv. 2024 Sep 14:2024.09.07.611823. Preprint. [Version 1] doi: 10.1101/2024.09.07.611823 (PMC11419055; doi:10.1101/2024.09.07.611823)

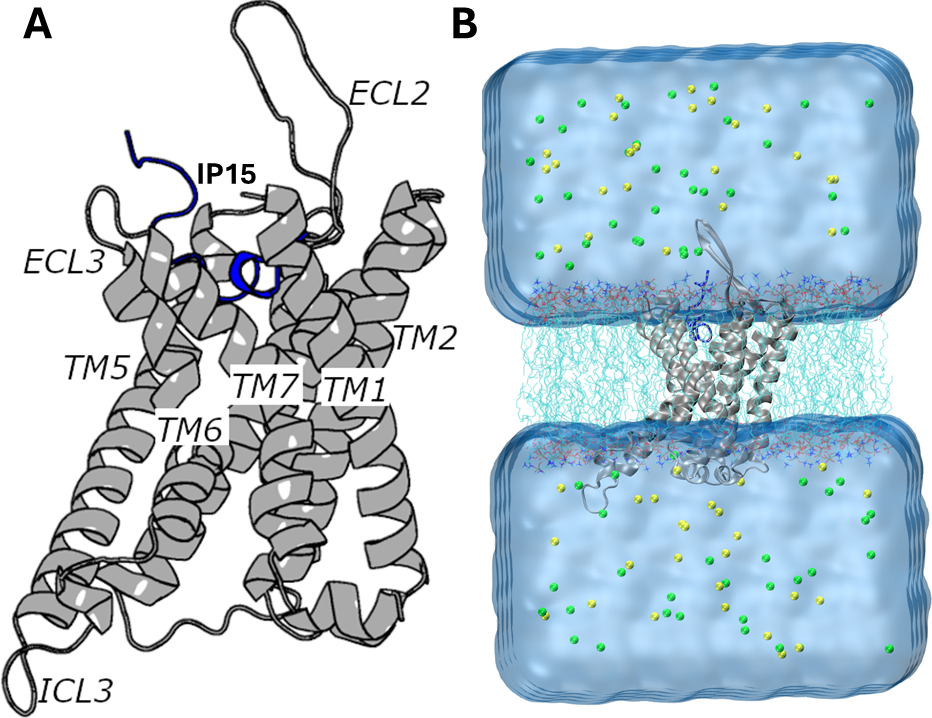

Supplement: Supplement 2 [file media-2.tif]

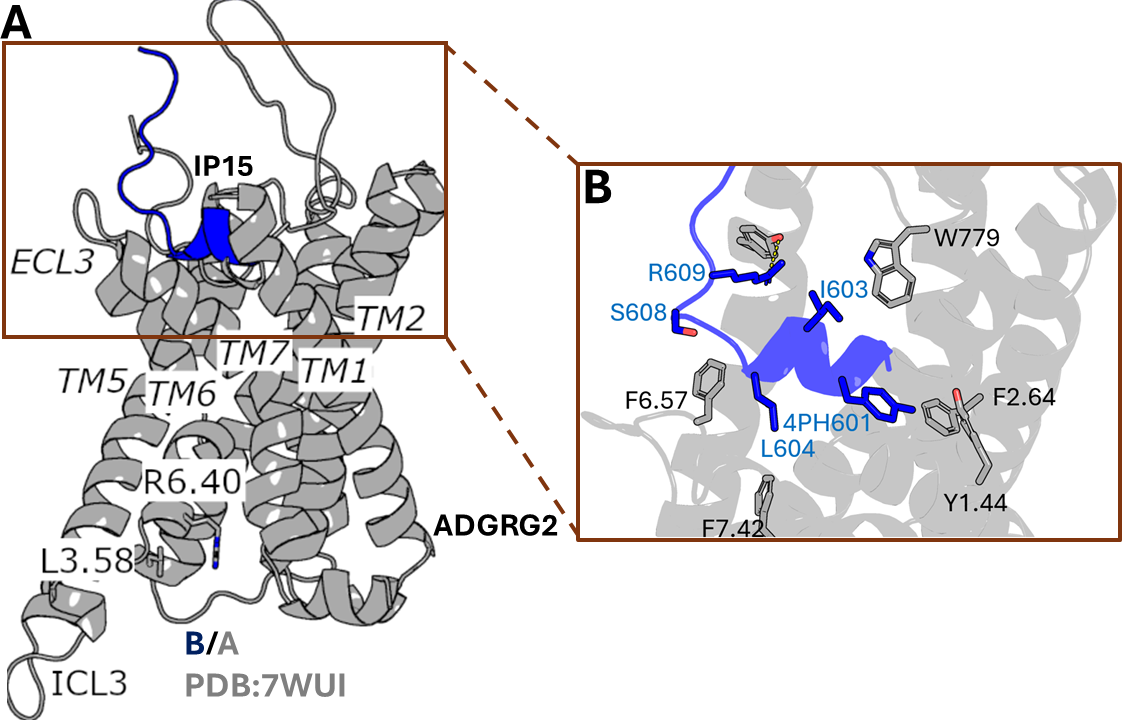

Supplement: Supplement 3 [file media-3.tif]

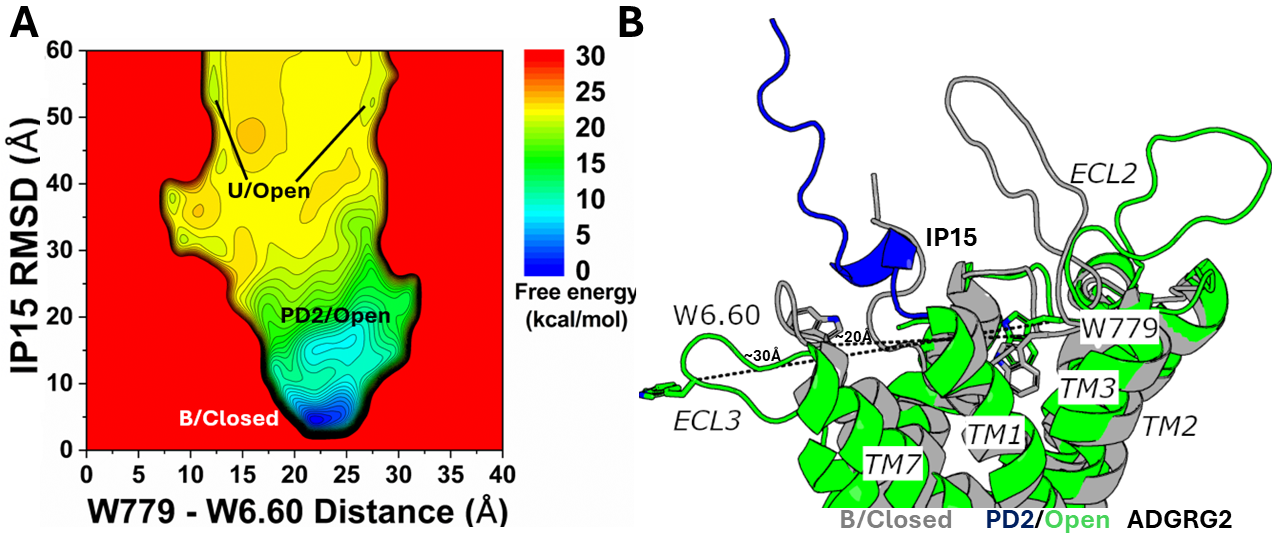

Supplement: Supplement 4 [file media-4.tif]

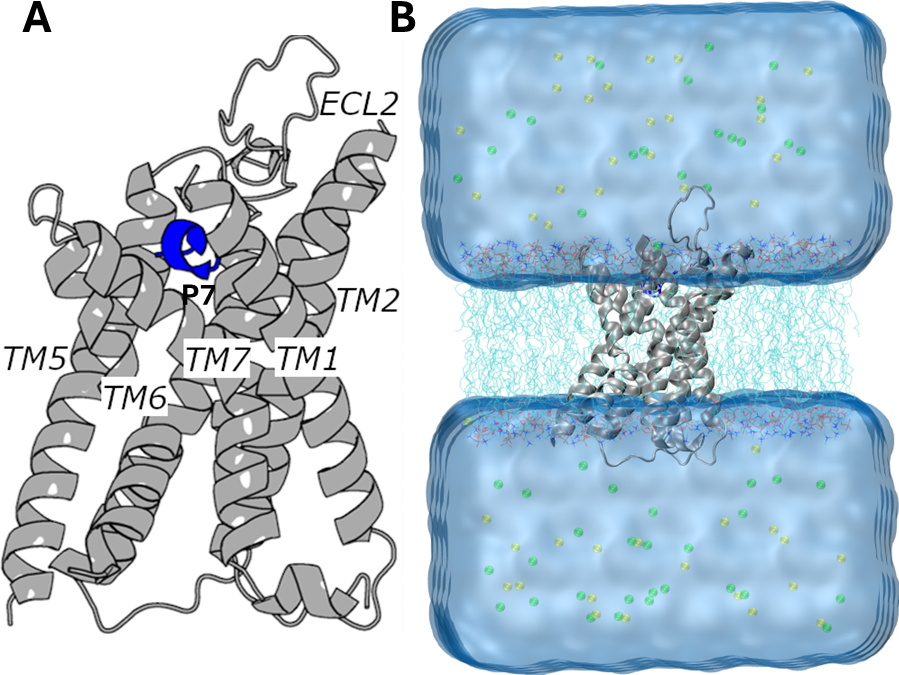

Supplement: Supplement 5 [file media-5.tif]

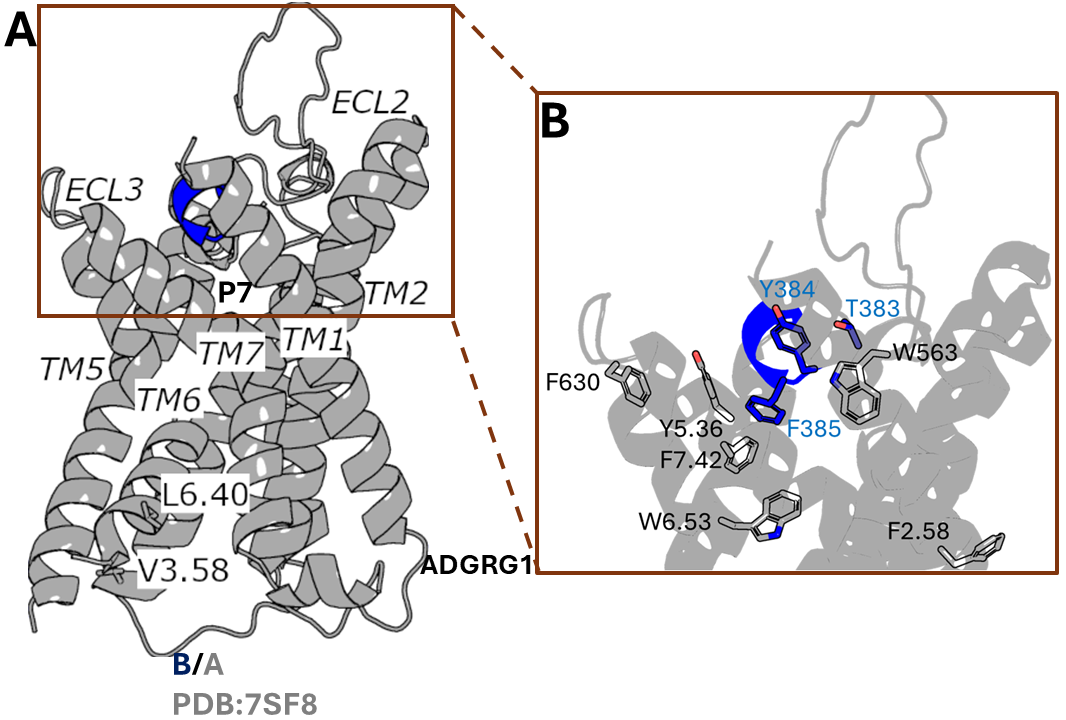

Supplement: Supplement 6 [file media-6.tif]
